# S140. VOICE-SELECTIVE FORWARD MODEL ABNORMALITIES IN NONCLINICAL VOICE HEARERS

**DOI:** 10.1093/schbul/sby018.927

**Published:** 2018-04-01

**Authors:** Ana Pinheiro, Michael Schwartze, Sonja Kotz

**Affiliations:** 1 University of Lisbon; 2 Maastricht University

## Abstract

**Background:**

Auditory verbal hallucinations (AVH) are one of the cardinal symptoms of psychosis but they are also present in 6–13% of individuals in the general population. Impaired predictive internal forward modelling has been proposed to underlie the experience of AVH in psychotic patients, but it remains unclear whether similar abnormalities are also present in nonclinical voice hearers. The current study sought to answer the question of whether and how hallucination predisposition modulates sensory prediction of tones and voices using event-related potentials (ERP) of the electroencephalogram (EEG).

**Methods:**

Participants with low (n=15) and high (n=17) hallucination predisposition, classified based on their Launay-Slade Hallucination Scale (LSHS) scores, were tested in an auditory task involving presentation of self-triggered and externally triggered tonal or own voice stimuli.

**Results:**

Participants with low and high hallucination predisposition displayed comparable N1 suppression effects to self-triggered tones (no significant group effect – p>.05) but the latter displayed enhanced N1 (group x condition x ROI interaction - F(4, 120)=7.971, p<.001) and reduced P2 (group x condition x ROI interaction - F(4, 120)=5.626, p<.001) responses to their self-triggered voice. Further, pre-stimulus alpha power was enhanced for self-triggered voices compared to tones in individuals with high hallucination predisposition (group x stimulus type interaction - F(1, 30)=4.479, p=.043). Anomalies in forward modelling were specifically associated with LSHS auditory hallucination scores (r=-471, p=.003).

**Discussion:**

Together, these findings suggest that altered forward modelling of one’s own voice is core to AVH. These results also provide partial support for the continuum model of psychosis, suggesting that psychotic symptoms form a continuum in the general population. A voice-specific, rather than a generalized, forward model dysfunction may explain why hallucinated voices are the most common type of auditory hallucinations.

